# ***β*** Common Receptor Mediates Erythropoietin-Conferred Protection on OxLDL-Induced Lipid Accumulation and Inflammation in Macrophages

**DOI:** 10.1155/2015/439759

**Published:** 2015-05-25

**Authors:** Tzong-Shyuan Lee, Kuo-Yun Lu, Yuan-Bin Yu, Hsueh-Te Lee, Feng-Chuan Tsai

**Affiliations:** ^1^Department of Physiology, National Yang-Ming University, Taipei 11221, Taiwan; ^2^Brain Research Center, National Yang-Ming University, Taipei 11221, Taiwan; ^3^Genome Research Center, National Yang-Ming University, Taipei 11221, Taiwan; ^4^Division of Hematology and Oncology, Taipei Veterans General Hospital, Taipei 11221, Taiwan; ^5^Institute of Anatomy and Cell Biology, National Yang-Ming University, Taipei 11221, Taiwan

## Abstract

Erythropoietin (EPO), the key factor for erythropoiesis, also protects macrophage foam cells from lipid accumulation, yet the definitive mechanisms are not fully understood. *β* common receptor (*β*CR) plays a crucial role in the nonhematopoietic effects of EPO. In the current study, we investigated the role of *β*CR in EPO-mediated protection in macrophages against oxidized low-density lipoprotein- (oxLDL-) induced deregulation of lipid metabolism and inflammation. Here, we show that *β*CR expression was mainly in foamy macrophages of atherosclerotic aortas from apolipoprotein E-deficient mice. Results of confocal microscopy and immunoprecipitation analyses revealed that *β*CR was colocalized and interacted with EPO receptor (EPOR) in macrophages. Inhibition of *β*CR activation by neutralizing antibody or small interfering RNA (siRNA) abolished the EPO-conferred protection in oxLDL-induced lipid accumulation. Furthermore, EPO-promoted cholesterol efflux and upregulation of ATP-binding cassette (ABC) transporters ABCA1 and ABCG1 were prevented by pretreatment with *β*CR neutralizing antibody or *β*CR siRNA. Additionally, blockage of *β*CR abrogated the EPO-conferred anti-inflammatory action on oxLDL-induced production of macrophage inflammatory protein-2. Collectively, our findings suggest that *β*CR may play an important role in the beneficial effects of EPO against oxLDL-elicited dysfunction of macrophage foam cells.

## 1. Introduction

Erythropoietin (EPO) was first identified in kidney to function as a crucial humoral regulator in erythropoiesis by promoting proliferation, differentiation, and survival of erythroid precursors [1, 2]. Based on this biological activity, EPO has been used for the treatment of selected red blood disorders in patients [2, 3]. Now, it is evident that EPO and its receptor, EPOR, exhibit a widespread distribution among a variety of organs in addition to kidney [1, 3, 4]. Recently, a large body of additional studies demonstrates that EPO also has several extrahematopoietic protective effects on the cardiovascular system, nervous system, and immunity [4–8]. For instance, EPO enhances reendothelialization after vascular injury and protects the endothelial cells (ECs) from hypoxia-induced apoptosis [7, 9, 10]. EPO promotes neuron development, prevents cell death of neurons, and improves learning and memory [11–13]. Furthermore, EPO retards the progression of atherosclerosis in chronic kidney disease patients [12]. We previously reported that EPO inhibits the formation of macrophage foam cells by promoting the efficacy of cholesterol efflux [13]. Emerging evidence suggests that *β* common receptor (*β*CR), a shared receptor subunit of interleukin-3 (IL-3), IL-5, and granulocyte-macrophage colony stimulation factor (GM-CSF) receptors, mediates several nonhematopoietic effects of EPO in various types of cells [14–18]. Blockage of *β*CR activity by neutralizing antibody prevents the EPO-mediated endothelial nitric oxide synthase (eNOS) activation and nitric oxide (NO) production in ECs [17]. Genetic deletion of *β*CR abrogates the protective effect of EPO in spinal cord injury mouse model [18]. Although the implication of EPO in deregulation of cholesterol metabolism of foam cells has been defined, the role of *β*CR in pathophysiology of macrophage foam cells is poorly understood. To this end, further investigation delineating the role of *β*CR in EPO-mediated benefits in the lipid metabolism and inflammation of foam cells is warranted.

Complications of atherosclerosis are the leading cause of death in developed and developing countries. Deregulation of cholesterol metabolism is the most critical factor for the development of atherosclerosis, which leads to the deposition of excessive cholesterol within vessel wall and persistent vascular inflammation [19, 20]. Macrophage foam cell-mediated regulation of cholesterol metabolism and inflammation are central events in the initiation and progression of atherosclerosis [21–24]. Excessive lipid accumulation inside macrophage foam cells is mainly due to uncontrolled uptake of oxidized low-density lipoprotein (oxLDL) or impaired cholesterol efflux in macrophages [23, 24]. Intracellular lipid contents of macrophage foam cells are tightly regulated by scavenger receptors (SRs) and cholesterol efflux transporters. In macrophage foam cells, SRs such as SR-A and CD36 are responsible for internalizing oxLDL [25, 26]. In contrast, the efflux of intracellular cholesterol to high-density lipoprotein (HDL) is mediated by cholesterol efflux transporters including SR-BI and ATP-binding cassette (ABC) transporters [27–29]. Emerging evidence suggests that lowers lipid accumulation and inflammation by upregulating ABC transporters can be a therapeutic strategy for treating or preventing atherosclerosis [30–35]. Our previous study shows EPO upregulates the expression of ABCA1 and ABCG1 without affecting expression of SR-BI, SR-A, and CD36 in macrophages [13]. However, how *β*CR involves in EPO-elicited reduction in oxLDL-induced lipid accumulation and inflammation of macrophage foam cells remains to be explored.

Given the importance of *β*CR in EPO-conferred nonhematopoietic protection, in the present study, we aimed to investigate the role of *β*CR in EPO-conferred protection from the formation of macrophage foam cells in bone-marrow-derived macrophages (BMDMs). This study was conducted, firstly, to investigate the protein expression of *β*CR in atherosclerotic aortas and macrophages; secondly, to delineate the role of *β*CR in the suppression of EPO on cholesterol accumulation; and thirdly, to explore the role of *β*CR in oxLDL-elicited inflammation in macrophages. We found that the inhibition of *β*CR abolished the EPO-conferred protection from oxLDL-induced deregulation of lipid metabolism and inflammatory response in macrophages.

## 2. Materials and Methods

### 2.1. Reagents

Recombinant mouse erythropoietin (rhEPO), macrophage colony stimulating factor (M-CSF), and macrophage inflammatory protein-2 (MIP-2) ELISA kits were obtained from R&D Systems (Minneapolis, MN, USA). Minimum essential medium *α* (MEM*α*) and RPMI 1640 medium were purchased from Gibco Life Technology (Karlsruhe, Germany). Goat anti-EPO, rabbit anti-EPOR, anti-*β*CR antibodies, control small interfering RNA (siRNA), and *β*CR siRNA were obtained from Santa Cruz Biotechnology (Santa Cruz, CA, USA). Mouse anti-ABCA1 antibody was obtained from Abcam (Cambridge, MA, USA). Rabbit anti-ABCG1 was from Novus Biologicals (Littleton, CO, USA). Mouse anti-*α*-tubulin antibody, apolipoprotein AI (apoAI), high-density lipoprotein (HDL), 3,3-diaminobenzidine (DAB), bovine serum albumin (BSA), and human LDL were purchased from Sigma Chemical (St. Louis, MO, USA). NBD-cholesterol was from Cayman Chemical (Ann Arbor, MI, USA). Cholesterol and triglyceride assay kits were from Randox (Crumlin Co., Antrim, UK).

### 2.2. Immunohistochemical Assessment

Formalin-fixed, paraffin-embedded tissue blocks were cut into 8 *μ*M sections. Samples were mounted on positively charged glass microscope slides. Sections were deparaffinized, rehydrated, and then covered 3% H_2_O_2_ for 10 min. After incubation with blocking BSA for 30 min, slides were incubated with one of the following primary antibodies for 1 h at 37°C: EPOR or *β*CR. Then, sections were incubated with second antibodies appropriate for primary antibodies for 1 h at 37°C. Antigenic sites were visualized by the addition of DAB. Slides were counterstained with hematoxylin. Negative control slides were stained using the same procedure, omitting the primary antibody.

### 2.3. Cell Culture

Bone-marrow-derived macrophages (BMDMs) were prepared as previously described [36]. Briefly, SJL mice were killed by carbon dioxide exposure. Mononuclear cells were obtained from the femurs and tibias and were harvested by Percoll (1.073 g/cm^3^) density gradient centrifugation. The cells were then seeded in MEM*α* supplemented with 50 ng/mL M-CSF, 10% FBS, and penicillin (100 U/mL)/streptomycin (100 *μ*g/mL) at 37°C in humidified air with 5% CO_2_ for 5 days until cell populations became 80% confluent. All animal experiments were approved by the Animal Care and Utilization Committee of the National Yang-Ming University, Taiwan.

### 2.4. Low-Density Lipoprotein Modification

The oxLDL was prepared as described previously [37]. Briefly, LDL was exposed to 5 *μ*M CuSO_4_ for 24 h at 37°C and Cu^2+^ was then removed by extensive dialysis. The extent of modification was determined by the measurement of thiobarbituric acid-reactive substances (TBARs). The oxLDL containing approximately 30–60 nmol of TBARs as malondialdehyde equivalents per milligrams of LDL protein was used for experiments.

### 2.5. Oil-Red O Staining

After fixation in 4% paraformaldehyde, cells were stained by oil-red O staining for 30 min. Hematoxylin was used as counterstaining. The density of lipid content was evaluated by alcohol extraction after oil-red O staining.

### 2.6. Cholesterol and Triglyceride Measurement

Cellular cholesterol and triglycerides were extracted by the use of hexane/isopropanol (3/2, v/v). After expelling cellular debris, the supernatant was dried under nitrogen flush. Cholesterol and triglycerides content were measured by the use of cholesterol and triglyceride assay kits.

### 2.7. Quantitative Real-Time RT-PCR

Total cellular RNA was extracted by the use of total RNA reagent. A 5 *μ*g amount of total RNA was converted to complementary DNA (cDNA) by the use of reverse transcriptase (Fermentas, MD, USA). The resulting cDNA was used as templates for quantitative real-time RT-PCR (qPCR) with the TaqMan probe-based real-time quantification system (Applied Biosystems, Foster, CA, USA). The mRNA levels were normalized to those of GAPDH and expressed as fold change over controls.

### 2.8. Western Blot

Cells were rinsed twice with ice-cold PBS and then lysed with PBS containing 1% Triton X-100, 0.1% SDS, 0.5% sodium deoxycholate, 1 *μ*g/mL leupeptin, 10 *μ*g/mL aprotinin, and 1 mM phenylmethylsulfonyl fluoride on ice. After sonication for 15 s, crude extracts were subjected to centrifugation at 12000 ×g for 5 min at 4°C. The supernatants were collected as cell lysates. All protein concentrations were determined by a protein assay (Bio-Rad Laboratories, Richmond, CA, USA). Aliquots (50 *μ*g) of cell lysates or nuclear extracts were separated and electrophoresed on 8 or 12% SDS-polyacrylamide gel and then transblotted onto the Immobilon-P membrane (Millipore, Bedford, MA, USA). After being blocked with 5% skim milk in Tween/PBS, blots were incubated with various primary antibodies and then incubated with HRP-conjugated secondary antibodies. The protein bands in the blots were detected using enhanced chemiluminescence kit and quantified by ImageQuant 5.2 software (Healthcare Bio-Sciences, Philadelphia, PA, USA).

### 2.9. Statistical Analysis

Results are presented as mean ± SEM from 5 independent experiments. Mann-Whitney test was used to compare 2 independent groups. Kruskal-Wallis followed by the Bonferroni post hoc analysis was used to account for multiple testing. Statistical analysis involved SPSS v18.0 (SPSS Inc., Chicago, IL, USA). A *P* < 0.05 was considered statistically significant.

## 3. Results

### 3.1. *β*CR Is Expressed in Atherosclerotic Lesions and Macrophages

**Figure 1:**
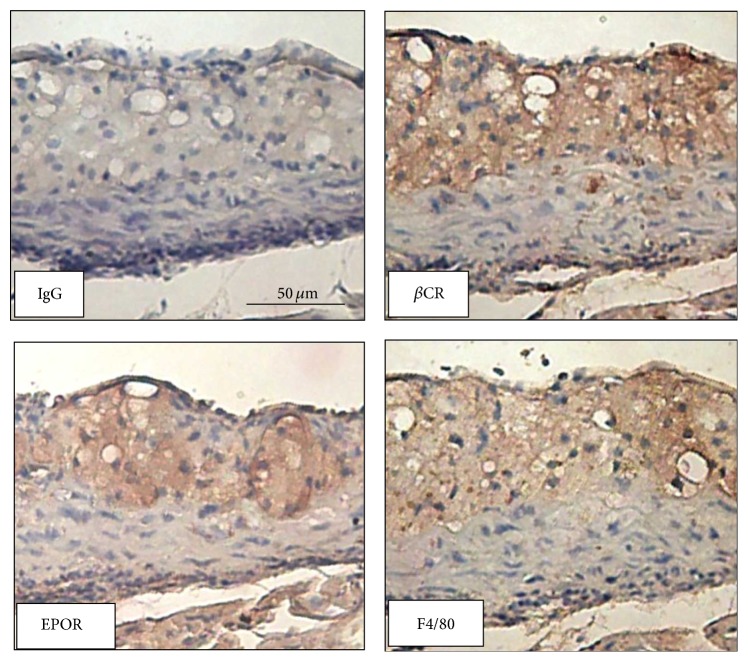
Expression of *β*CR in atherosclerotic lesions of apoE^−/−^ mice. Immunohistochemistry with IgG, anti-*β*CR, anti-EPOR, or anti-F4/80 was performed and then recognized by the corresponding horseradish peroxidase-conjugated secondaryantibody on aortic specimens of apoE deficient mice. Hematoxylin was used as counterstaining.

**Figure 2:**
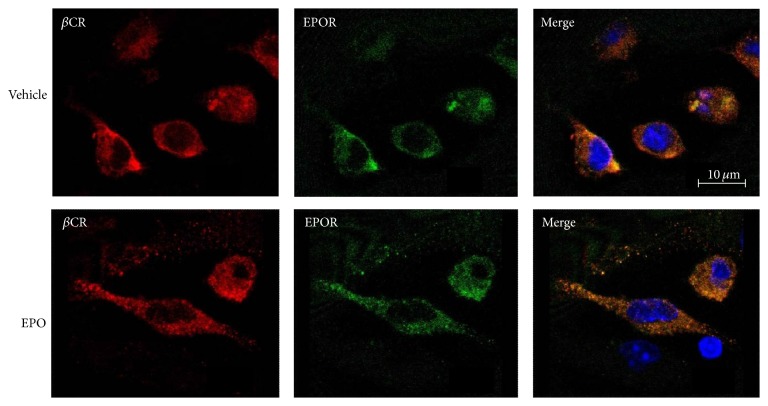
*β*CR colocalizes with erythropoietin receptor (EPOR) in macrophagesbone-marrow-derived macrophages were treated with vehicle (PBS) or EPO (5 U/mL) for 10 min. Cells were fixed and immunostained with anti-*β*CR or anti-EPOR Ab for confocal microscopy examination. Nuclei were stained with DAPI.

To study the possible role of *β*CR in atherogenesis, we first investigated the expression of *β*CR in atherosclerotic lesions. Immunohistochemical staining for *β*CR demonstrated that *β*CR was colocalized with EPOR and its positive signals are restricted mainly to areas of macrophages in atherosclerotic lesions of apoE^−/−^ mice (Figure 1). Results of confocal microscopy further confirm that *β*CR was detectable in BMDMs (Figure 2). These results imply that *β*CR may play a role in the biology of macrophage foam cells and atherogenesis.

### 3.2. EPO Does Not Affect the Dimerization of *β*CR and EPOR in Macrophages

**Figure 3:**
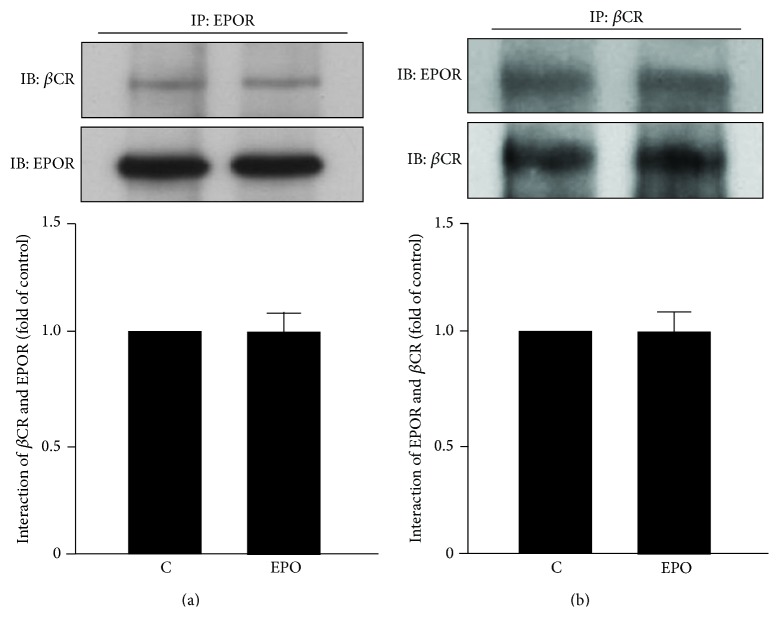
EPO treatment does not affect the interaction of *β*CR and EPOR in macrophages. Macrophages were treated with or without EPO (5 U/mL) for 10 min. Cellular lysates were immunoprecipitated (IP) with (a) anti-EPOR Ab or (b) anti-*β*CR Ab and then were immunoprobed with anti-EPOR or anti-*β*CR Ab by immunoblotting (IB). Data are mean ± SEM from 5 independent experiments.

It has been demonstrated that *β*CR/EPOR dimer is crucial for the nonhematopoietic protective effects of EPO [14–18]. We therefore examined whether EPO could alter the status of *β*CR/EPOR dimerization in macrophages. We found that treatment with EPO had no effect on the dimerization of *β*CR and EPOR (Figure 3).

### 3.3. *β*CR Mediates the EPO-Conferred Protection from Foam Cell Formation

**Figure 4:**
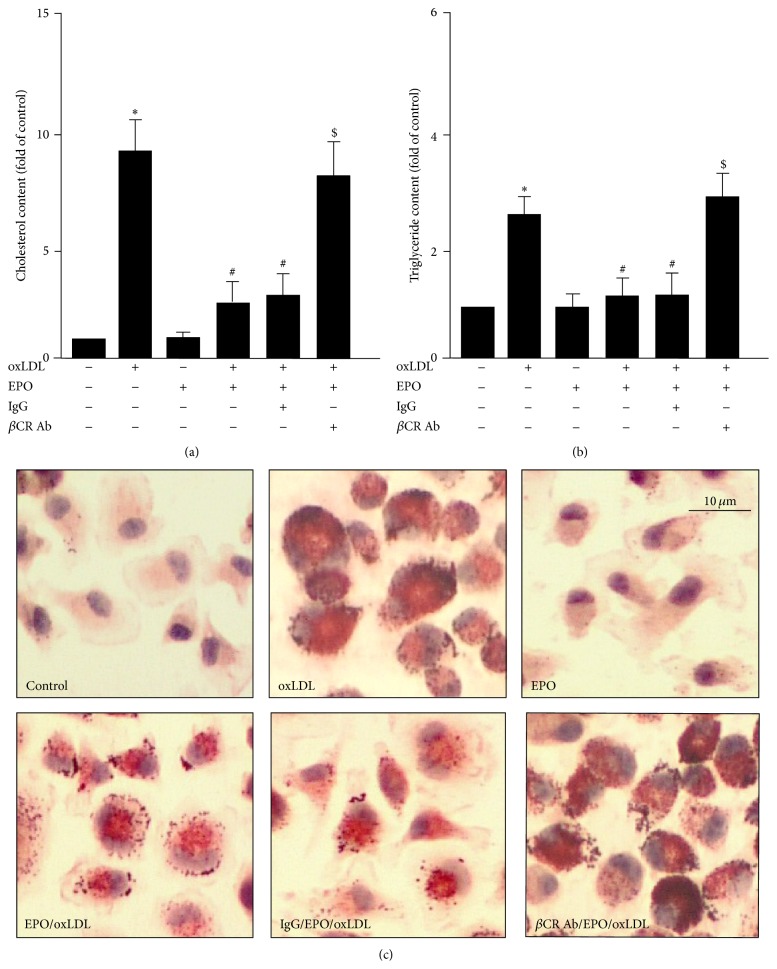
*β*CR neutralizing Ab diminishes the EPO-mediated protection of oxLDL-induced lipid accumulation in macrophages. Bone-marrow-derived macrophages were pretreated with IgG (200 ng/mL) or *β*CR neutralizing Ab (200 ng/mL) for 2 h and then treated with EPO (5 U/mL) for 12 h, followed by oxLDL (50 *μ*g/mL) for additional 18 h. ((a) and (b)) The intracellular levels of cholesterol (a) and triglyceride (b) were extracted by hexane/isopropanol (3/2, v/v) and analyzed by colorimetric assay kits or (c) subjected to oil-red O staining. Cellular nuclei were stained with hematoxylin. Data is mean ± SEM from 5 independent experiments. ^*∗*^ *P* < 0.05 versus vehicle-treated group, ^#^ *P* < 0.05 versus oxLDL alone-treated group, ^$^ *P* < 0.05 versus oxLDL+EPO-treated group.

**Figure 5:**
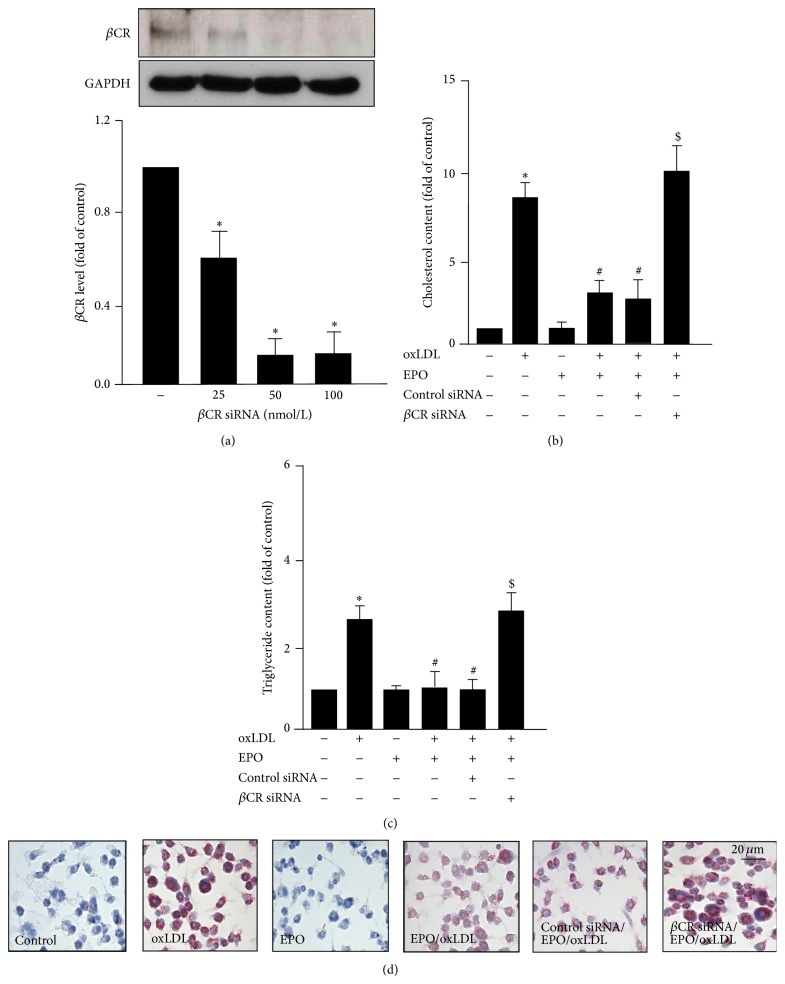
Knockdown of *β*CR expression ablates the EPO-mediated protection of oxLDL-induced lipid accumulation in macrophages. (a) Bone-marrow-derived macrophages were transfected with indicated concentrations of *β*CR siRNA (25, 50, and 100 nmole/L) for 24 h. Protein level of *β*CR was examined by western blot analysis. Cells were transfected with control siRNA (50 nmole/L) or *β*CR siRNA (50 nmole/L) for 24 h and then treated with EPO (5 U/mL) for 12 h, followed by oxLDL (50 *μ*g/mL) for additional 18 h. ((b) and (c)) The intracellular levels of cholesterol and triglyceride were extracted by hexane/isopropanol (3/2, v/v) and analyzed by colorimetric assay kits or (d) subjected to oil-red O staining. Cellular nuclei were stained with hematoxylin. Data is mean ± SEM from 5 independent experiments. ^*∗*^ *P* < 0.05 versus vehicle-treated group, ^#^ *P* < 0.05 versus oxLDL alone-treated group, ^$^ *P* < 0.05 versus oxLDL+EPO-treated group.

We have reported the beneficial effect of EPO on oxLDL-induced the lipid accumulation in macrophages [13]. We then determined the functional significance of *β*CR in EPO-mediated protection from foam cell formation in BMDMs. Loss of function of *β*CR by neutralizing Ab or knockdown gene expression demonstrated that EPO failed to prevent the foam cell formation in BMDMs as compared to the IgG- or control siRNA-treated BMDMs (Figures 4 and 5). These findings suggest that *β*CR plays a crucial role in the EPO-conferred protection in the formation of macrophage foam cells.

### 3.4. *β*CR Is Essential for the EPO-Elicited Promotion of Cholesterol Efflux in Macrophages

**Figure 6:**
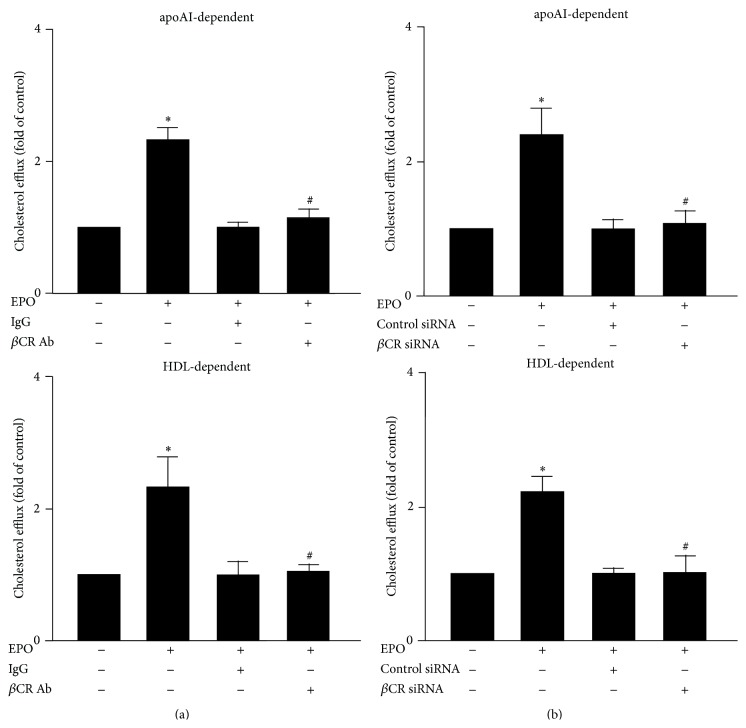
Inhibition of *β*CR activation abolishes the EPO-mediated promotion of cholesterol efflux in macrophages. Bone-marrow-derived macrophages were pretreated with IgG (200 ng/mL) or *β*CR neutralizing Ab (200 ng/mL) for 2 h (b) transfection with control siRNA (50 nmole/L) or *β*CR siRNA (50 nmole/L) for 24 h and then treated with EPO (5 U/mL) for 12 h, followed by NBD-cholesterol (1 *μ*g/mL) for additional 6 h in the absence or presence of apoAI (10 *μ*g/mL) or HDL (50 *μ*g/mL). The medium and cell lysates were collected for the measurement of fluorescence. The calculation of cholesterol efflux is defined as fluorescence in the medium relative to the total amount of fluorescence. Data are mean ± SEM from 5 independent experiments. ^*∗*^ *P* < 0.05 versus vehicle-treated group, ^#^ *P* < 0.05 versus EPO-treated group.

**Figure 7:**
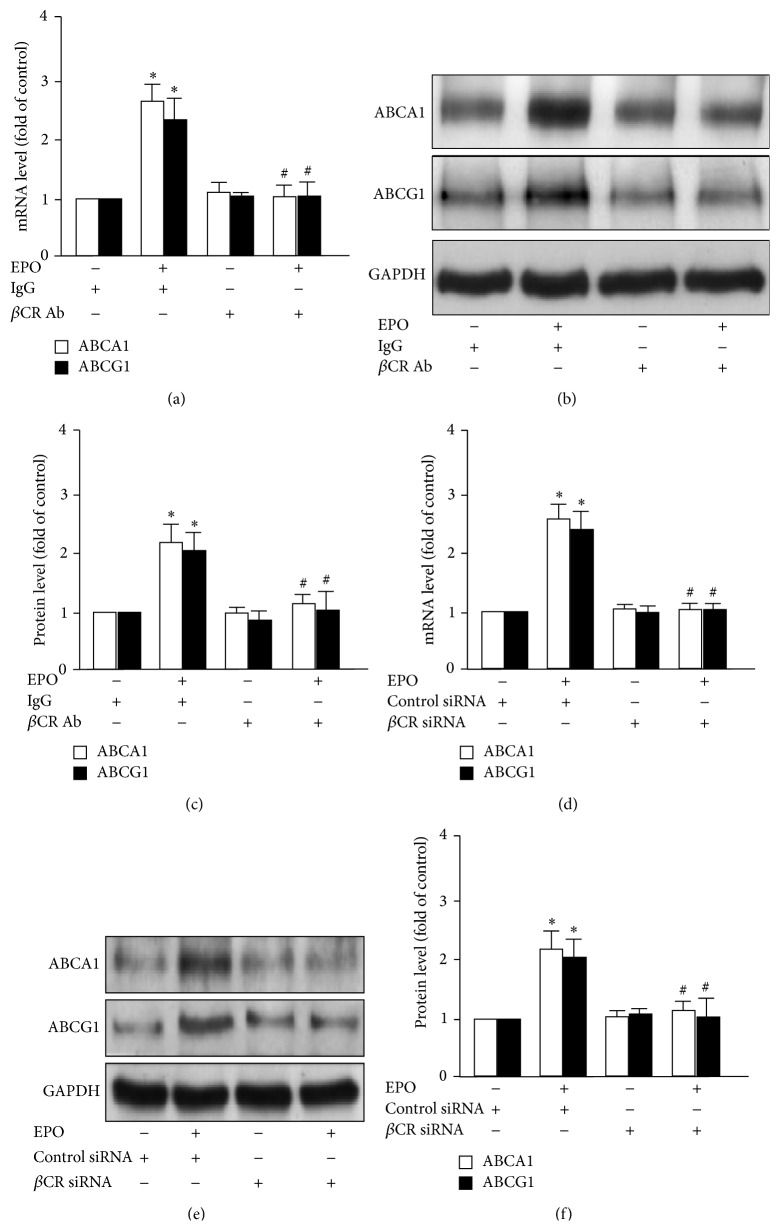
Blockage of *β*CR activation ablates EPO-induced upregulation of ABCA1 and ABCG1 transporters in macrophages. Bone-marrow-derived macrophages were pretreated with IgG (200 ng/mL) or *β*CR neutralizing Ab (200 ng/mL) for 2 h (b) transfection with control siRNA (50 nmole/L) or *β*CR siRNA (50 nmole/L) for 24 h and then treated with EPO (5 U/mL) for 12 h or 24 h. Cell were lyzed and lyates subjected to RT-PCR and western blot analysis for mRNA or protein expression of ABCA1, ABCG1, and GAPDH. Data is mean ± SEM from 5 independent experiments. ^*∗*^ *P* < 0.05 versus vehicle-treated group and ^#^ *P* < 0.05 versus EPO-treated group.

EPO is known to promote the ABC transporter-dependent cholesterol efflux in macrophages [13]. We then investigated the role of *β*CR in EPO-mediated increase in cholesterol efflux and expression of ABCA1 and ABCG1. Our data showed that treatment with *β*CR neutralizing Ab or *β*CR siRNA abolished EPO-stimulated apoAI- and HDL-dependent cholesterol efflux in BMDMs (Figure 6). In addition, the mRNA and protein expression of ABCA1 and ABCG1 induced by EPO were prevented under the condition with the loss of function of *β*CR (Figure 7). Collectively, these data suggest that the *β*CR is required for the EPO-induced suppression of intracellular lipid accumulation in macrophage foam cells.

### 3.5. *β*CR Is a Key Molecule for the Anti-Inflammatory Effect of EPO in OxLDL-Induced Inflammation

**Figure 8:**
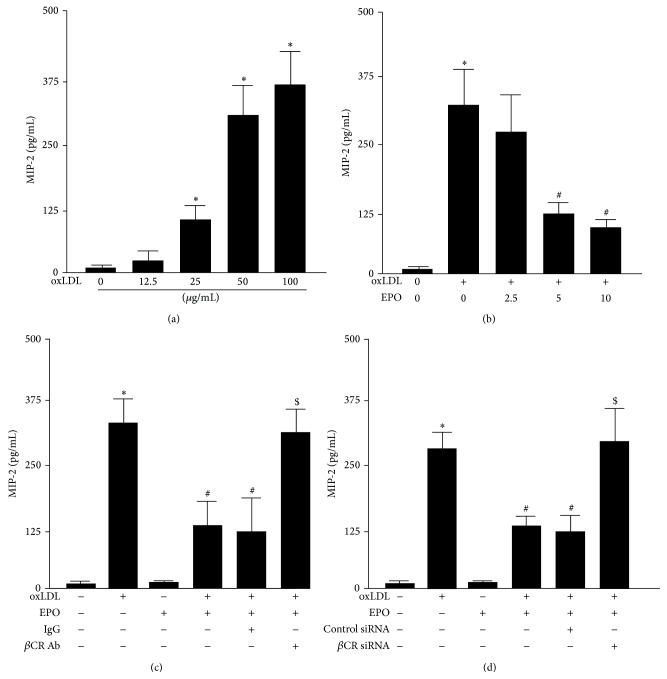
Inhibition of *β*CR activation prevents the protective effect of EPO against from the oxLDL-induced MIP-2 production in macrophages. (a) BMDMs were treated with indicated concentrations of oxLDL (12.5, 25, 50, and 100 *μ*g/mL) for 24 h. (b) Cells were treated with oxLDL (50 *μ*g/mL) in the absence or presence of indicated concentrations of EPO (2.5, 5, and 10 U/mL) for 24 h. (c) Cells were pretreated with IgG (200 ng/mL) or *β*CR neutralizing Ab (200 ng/mL) for 2 h or (d) transfection with control siRNA (50 nmole/L) or *β*CR siRNA (50 nmole/L) for 24 h and then treated with oxLDL (50 *μ*g/mL) and EPO (5 U/mL) for an additional 24 h. Levels of MIP-2 in cultured medium were analyzed by ELISA. Data are mean ± SD from 5 independent experiments. ^*∗*^ *P* < 0.05 versus vehicle-treated group, ^#^ *P* < 0.05 versus oxLDL alone-treated group, and ^$^ *P* < 0.05 versus oxLDL-treated group with EPO-treatment.

EPO is reported to have anti-inflammatory properties [38, 39]. We next examined the role of *β*CR in oxLDL-induced production of MIP-2 in BMDMs. Our data showed that MIP-2 production increased in a concentration-dependent manner with oxLDL treatment (Figure 8(a)). This oxLDL-increased production of MIP-2 in BMDMs was significantly attenuated by treatment with the EPO (Figure 8(b)). Moreover, pretreatment with *β*CR neutralizing Ab or *β*CR siRNA abolished EPO-provided anti-inflammatory effect on oxLDL-induced MIP-2 production (Figures 8(c) and 8(d)). These results suggest that *β*CR may be required for the anti-inflammatory action of EPO in the process of macrophage foam cell formation in response to oxLDL challenge.

## 4. Discussion

EPO, the key hormone for erythropoiesis, also has inhibitory effect on the formation of macrophage foam cells, yet the detailed molecular mechanisms are not fully understood. We investigated the role of *β*CR in EPO-mediated suppression on oxLDL-induced lipid accumulation and inflammation. Our results demonstrated that *β*CR may play an important role in the EPO-mediated cholesterol metabolism and attenuation in oxLDL-induced inflammation in BMDMs. We first validated that *β*CR was colocalized and interacted with EPOR in macrophages and in atherosclerotic lesions. Pretreatment with neutralizing antibody or siRNA for *β*CR abolished the EPO-conferred protection against oxLDL-induced lipid accumulation and abrogated the EPO-induced upregulation of ABC transporters and the resulting surge of cholesterol efflux. The same treatment towards *β*CR also rendered the EPO anti-inflammatory activity against oxLDL-induced MIP-2 production ineffective. Accordingly, these findings suggest that *β*CR plays a key role in the nonhematopoietic benefits of EPO and may have therapeutic value in the treatment of atherosclerosis and disorders of lipid metabolism.

Emerging evidence indicates the crucial influence of EPO on the regulation of physiological functions of cardiovascular system and the pathogenesis of cardiovascular diseases [7, 8, 17, 40–43]. Experimental data indicate that EPO maintains the integrity of endothelial cells through an eNOS-dependent mechanism, and deletion of EPO or EPOR leads to angiogenic defects in the vascular system during embryogenesis [7, 8, 17, 40, 41]. Moreover, EPO improves cardiac function by increasing blood flow and inhibiting myocyte apoptosis in ischemia rodent models [42, 43]. On the contrary, clinical observations demonstrate that the mortality and morbidity rate are elevated in patients with acute ischemia stroke or chronic kidney diseases when administrated with EPO [44, 45]. Yet, there is not sufficient evidence to explain how EPO elicit these unfavorable effects in these EPO-treated patients.

Notably, the profile of lipid or apolipoproteins in hemodialysis patients was changed with long-term treatment with EPO [12, 46, 47]. Moreover, Buemi et al. reported that administration with EPO significantly reduced the cholesterol ester content of atherosclerotic aortas in Watanabe heritable hyperlipidemia rabbits [48]. Although the detailed cellular and molecular mechanisms by which EPO regulates the lipid metabolism* in vivo* are unclear, our previous findings suggest that EPO can directly regulate the cholesterol metabolism of macrophage foam cells by promoting cholesterol efflux. In this study, we further confirmed this beneficial effect of EPO and provide new evidence regarding with the implication of *β*CR in this antiatherogenic property of EPO.

EPO was first identified as a hormone produced in the kidney that promotes the erythropoiesis in bone marrow [1, 3, 4]. EPO regulates erythropoiesis by binding to the classical, homodimeric complex of two EPO receptors (EPOR2) on erythroid progenitor cells [3, 4]. Notably, the nonhematopoietic protection of EPO in other types of cells is mediated by the binding of EPO to a heteromeric receptor complex consisting of two EPOR and two *β*CR (EPOR2-*β*CR2) [4, 8], which is in line with our current findings, confirming the interaction between EPOR and *β*CR in BMSMs. In the adult, EPO is typically produced by the kidney, and EPOR is mainly expressed by cells in bone marrow; however, the level of EPOR in nonerythroid cells was much lower than that in erythroid cells [1–8]. Moreover, the binding affinity of the cytoprotective EPOR2-*β*CR2 complex for EPO is significantly lower as compared to the affinity of erythropoietic EPOR2 complex [7, 8]. The expressed pattern of EPOR2-*β*CR2 and its affinity with EPO in nonerythroid cells may explain the observation that the activation of EPOR2-*β*CR2 by EPO required much higher concentrations of EPO compared to that of EPOR2 [7, 8].

In addition to the practice of EPO in the treatment of anemia and renal diseases [2, 3], emerging evidence suggests that EPO has other beneficial effects on nonerythroid tissues [4–8]. However, a major disadvantage in the use of EPO as a cytoprotective agent is its stimulated effect on erythropoiesis [7, 8], which may indirectly influence the therapeutic efficacy of EPO or increase the risk of cardiovascular adverse events [7, 8]. For instance, polycythemic transgenic mice overexpressing EPO have increased the whole blood viscosity and enhanced platelet activation [49], which may induce life threatening adverse effects such as thrombotic events on clinical application [7, 8, 50]. To avoid these off-target events during the last decades, EPO derivatives have been developed that only activate the EPOR2-*β*cR2 complex and do not stimulate erythropoiesis [51–54]. For instance, carbamylated EPO, a nonhematopoietic analogue of EPO, which can bind to EPOR2-*β*CR2, is reported to provide comparable neuronal and myocardial cytoprotection with EPO in experimental animal models [51, 52]. More recent, ARA290, a small synthetic peptide without erythropoietic properties, selectively binds to EPOR2-*β*CR2 complex, and has been shown to confer renoprotection in the renal ischemia/reperfusion model [53, 54]. Mechanistically, EPO and its EPO derivatives afford several beneficial effects including anti-inflammation and antiapoptosis, as well as promotion of NO bioavailability by activating in a cascade of Janus kinase-2 (JAK2) signaling pathway or Akt signaling pathway [7, 8, 53, 54]. Our previous studies showed that the effect of EPO on NO production of ECs is mediated through the activation *β*CR/JAK2/Akt/AMPK/eNOS signaling pathway [17, 55]. In this study, we indeed observed that inhibition of JAK2 activation or Akt by pharmacological inhibitors abrogated the EPO-mediated suppression on the oxLDL-induced lipid accumulation in macrophage foam cells (data not shown). Nevertheless, whether carbamylated EPO and ARA290 afford the comparable protection as EPO, from the formation of foam cells and atherosclerosis, remains in need for further investigations.

## 5. Conclusions

In conclusion, this study demonstrates a unique function of *β*CR in EPO-mediated cytoprotection from oxLDL-induced deregulation of lipid metabolism and inflammatory response in macrophages. Our findings provide advanced information in EPO-conferred protection and suggest that *β*CR may have therapeutic value in treating atherosclerosis-related cardiovascular diseases.
